# Clinical Predictors for Successful Uvulopalatopharyngoplasty in the Management of Obstructive Sleep Apnea

**DOI:** 10.1155/2013/290265

**Published:** 2013-09-19

**Authors:** Aamir Yousuf, Zafarullah Beigh, Raja Salman Khursheed, Aleena Shafi Jallu, Rafiq Ahmad Pampoori

**Affiliations:** Department of ENT and HNS, Government Medical College, Srinagar, Jammu and Kashmir 190001, India

## Abstract

*Objective*. To assess the clinical parameters for successful uvulopalatopharyngoplasty in the management of obstructive sleep apnoea syndrome documented with pre- and postoperative polysomnography. *Materials and Methods*. A study group of 50 patients diagnosed as having OSA by full night polysomnography were assessed clinically and staged on basis of Friedman staging system. BMI and neck circumference were considered, and videoendoscopy with Muller's maneuver was done in all to document the site of obstruction. The study group divided into surgical and nonsurgical ones. Twenty-two patients out of fifty were then selected for uvulopalatopharyngoplasty. The selection of surgical group was done primarily on basis of clinical parameters like neck circumference, Friedman stage of the patient and site, and/or level of obstruction of patient. Postoperative polysomnography was done six months after surgery to document the change in AHI score. *Result*. The study group consists of fifty patients with mean age of 44.4 ± 9.3 years. UPPP was done in twenty-two, and the result of the surgery as defined by 50% reduction in preoperative AHI with postoperative AHI < 20/h was seen to be 95.2%. Postoperative change in AHI done after 6-month interval was seen to be statistically significant with *P* value < 0.001. *Conclusion*. UPPP is ideal option for management of obstructive sleep apnoea syndrome in properly selected patients on the basis of Friedman stage and site of obstruction detected by videoendoscopy with Muller's maneuver.

## 1. Introduction

Obstructive sleep apnea (OSA) is a common condition, affecting 4% of men and 2% of women [1]. Currently the condition is diagnosed by history, physical examination, imaging studies, and polysomnography. Common symptoms of the condition have limited predictive value in identifying patients with OSA. The upper airway is the main anatomical site responsible for OSA. Clinical examination may point to severe retrognathia, hypertrophic tonsils, macroglossia and redundant pillars, elongated uvula, and a crowded oropharynx [2]. Endoscopic investigations have been performed in awake as well as in sleeping patients, with the pharynx in relaxed or active states, but their predictive value remains limited, both for diagnostic purposes and for identifying patients who may benefit from surgery [3]. The otolaryngologist has the unique opportunity to examine the palate, pharynx, and neck of the patient and suspect OSA when appropriate. Diagnosis of a disease is based on clinical symptoms and physical findings and is corroborated by laboratory examinations. Polysomnography remains the standard in the diagnosis of sleep-related breathing disorders [2, 4]. Continuous positive airway pressure (CPAP), a technique that pneumatically supports the upper airway, is a therapeutic mainstay for OSA, other options for patients with OSA, including risk factor modification such as weight loss, oral appliances that advance the mandible or tongue during sleep, or a variety of surgical procedures to bypass or expand the upper airway [5]. The most common surgical procedure performed for OSA is uvulopalatopharyngoplasty (UPPP) Introduced by Fujita et al. in 1981; UPPP involves tonsillectomy (if not previously performed), trimming and reorientation of the posterior and anterior tonsillar pillars, and excision of the uvula and posterior palate. Often, UPPP is combined with other nasopharyngeal or oropharyngeal procedures. The reported success of UPPP as a treatment of OSA is between 16% and 83%, depending on the definition of a positive outcome and selection of patients. Some authors have defined surgical success or cure after UPPP as a 50% reduction in the AHI, whereas others combine this criterion with an absolute AHI of 20 or less [3, 6–8]. 

## 2. Materials and Methods

 This study was conducted in Department of Otorhinolaryngology and Head Neck Surgery, Government Medical College Srinagar, Jammu and Kashmir, India, from January 2010 to June 2011 and was approved by institutional ethics committee. Any patient who came to our department directly or had been referred from other centres with one or more complaints of excessive daytime somnolence (EDS), snoring, or observed apnoea was identified as high risk and underwent full assessment. The study group of total 50 patients was selected among those most high risk patients for OSAS and was analysed thoroughly and properly diagnosed as obstructive sleep apnoea using full night polysomnography. The sleep study (in hospital full night polysomnography) of all the patient was done in order to objectively quantify any sleep apnoea using Embletta Gold device, and the data was analysed by using Remlogic software. The parameters considered were electroencephalography, electrocardiography, abdominal movements, thoracic movements, snore nasal pressure by nasal thermistor, Spo2 level (pulse oximeter), Pulse rate, Body position, and flow pressure (nasal cannula). On the basis of these parameters apnea, hypopneas, snore level, and oxygen deasturation level were noticed, and patients were classified on the basis of their AHI (apnea/hypopnea index). The various events/indexes of sleep-related breathing disorders, apnea, is defined as reduction in airflow greater than ≥90% as recorded by oronasal thermistors or nasal pressure cannulas lasting ≥10 sec. Hypopnea is defined as reduction in airflow ≥30% as recorded by nasal pressure cannulas or alternatively by induction of plethysmography or oronasal thermistors lasting ≥10 sec with reduction in saturation at least ≥4% from baseline SpO2% prior to the event. Apnea-hypopnea index (AHI) is defined as the number of apneas and hypopneas per hour of sleep, confirmed by electroencephalogram (EEG) [2]. All the essential anthropometric measurements like neck circumference and body mass index were tabulated. The clinical assessment of the upper airway was done for any abnormality that could contribute to airway narrowing, such as a deviated nasal septum or a small oropharyngeal airway and tonsil size, and tongue/palatal position was considered [7, 9]. The Friedman tongue position is based on visualization of structures in the mouth with the mouth open widely without protrusion of the tongue. Palate Grade I allows the observer to visualize the entire uvula and tonsils. Palate Grade II allows visualization of the uvula but not the tonsils. Palate Grade III allows visualization of the soft palate but not the uvula. Palate Grade IV allows visualization of the hard palate. All the patients were then staged on the basis of Friedman staging system that includes tonsil size, Friedman tongue position, and BMI of patients. In all patients videoendoscopy with Muller's maneuver was done, and site of obstruction was documented. Videoendoscopy was done using fiberoptic laryngoscope, and the patients were explained to inspire forcibly against closed mouth and nose, and collapse of upper airway was documented at retropalatal, retrolingual, and hypopharyngeal levels [3, 10]. The study group of fifty was then divided into surgical and nonsurgical groups. Twenty-two patients out of fifty were selected for uvulopalatopharyngoplasty. The selection of surgical group was done primarily on the basis of clinical parameters like neck circumference, BMI and Friedman stage of the patient, and site and/or level of obstruction of the patient [10, 11]. Patients with Friedman stage I and II along with unilevel obstruction mostly at retropalatal level and with less neck circumference were selected for surgery. 

## 3. Results

**Figure 1:**
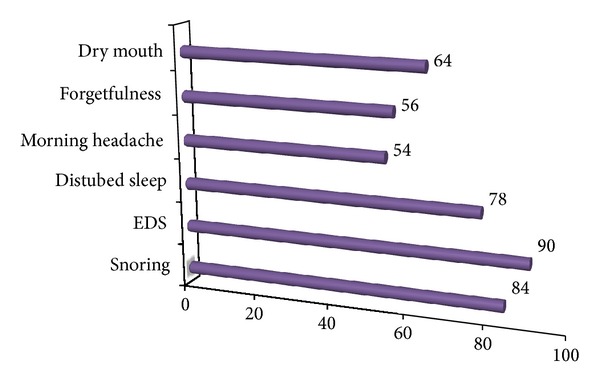
Distribution of presenting complaints.

**Figure 2:**
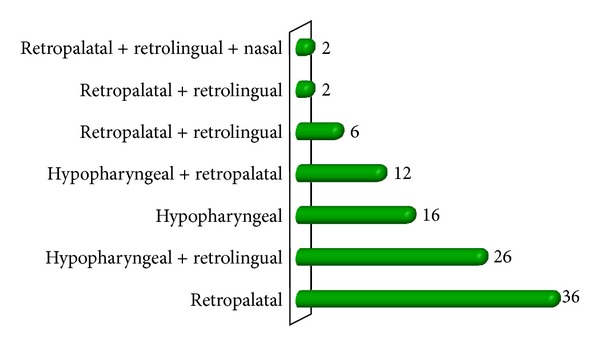
Site of obstruction seen by videoendoscopy with Muller's maneuver.

**Figure 3:**
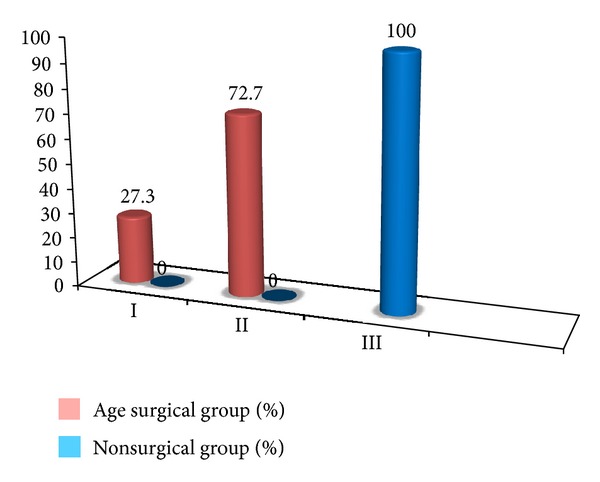
Comparison of surgical and nonsurgical group of patients on the basis of friedman stage.

**Figure 4:**
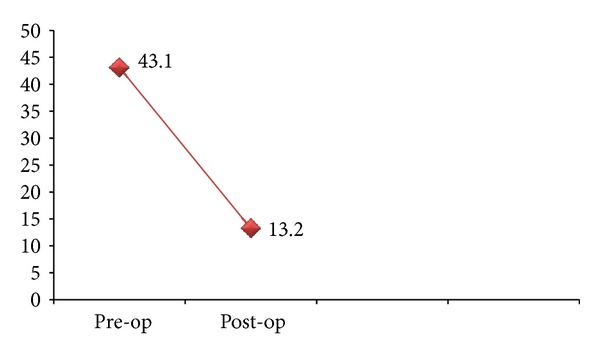
Preoperative and Postoperative AHI of surgical group.

The study group comprises of total fifty patients of mean age 44.4 ± 9.3 years with 56% males and 44% females Table 1. The most common presenting complaints of our patients were snoring seen in 84% and excessive day time sleepiness seen in 90% (Figure 1). All the patients were thoroughly evaluated, and neck circumference and body mass index (BMI) were considered. The neck circumference ranges from 24 cm to 42 cm with mean 36.6 cm, and the BMI ranges from 27 kg/m^2^ to 40 kg/m^2^ with mean 34.7 kg/m^2^. The mean AHI of all the patients was 53/h that ranges from 22 to 81/h. In our study group all the fifty patients were grouped on the basis of Friedman tongue position as I, II, III, and IV with 0%, 28%, 46%, and 26%, respectively. On basis of tonsil size of patients, it was seen that 30% of the patients had Grade 2 and 26% and 24% patients had Grade 1 and grade 3 tonsillar enlargement, respectively, and grade 0 was seen in 12% and grade 4 in 8% of patients. Grouping together these characteristics, all the patients were graded on Friedman staging system as stage I, II, and III. Friedman stage I constitutes 12%, stage II, 32%, and stage III, 56% of patients. For determining the exact site of obstruction in study group patients, videoendoscopy with Mueller's maneuver was done (Figure 3). In 36% of the patients only retropalatal obstruction was seen and in 16% only hypopharyngeal. The rest of the patients have multilevel obstruction with 26% having hypopharyngeal and retrolingual and 12% with hypopharyngeal and retropalatal. 6% of the patients had retropalatal and retrolingual; retropalatal, retrolingual, and nasal in 2%; and retropalatal, nasal in 2% of patients as shown in Table 2 and Figure 2. The degree of collapse of upper airway was graded as 1+ minimal collapse, 2+ is 50% collapse, 3+ is 75% collapse, and 4+ is obliteration of the airway. The study group of fifty patients was then divided into surgical and nonsurgical groups. The surgical group selected was entirely on clinical parameters like neck circumference, BMI, tonsil grade, tongue position, and level of upper airway collapse. There was significant (*P* value < 0.001) difference of these parameters among surgical and nonsurgical groups of patients as shown in Table 3. On the basis of tonsil grade criteria, the surgical group was selected with higher grade of tonsil (54.5% had Grade 3 and 27.3% had Grade 2); lesser tonsil grade patients were kept in nonsurgical group (Grade 1 in 46.4% and Grade 2 in 32.1%) as shown in Table 4. This was seen to be statistically significant with *P* value of <0.001. On the basis of Friedman tongue position, the patients with lower FTP were selected for surgical group (Grade 2,54.5% and Grade 3,45.5%) as compared to nonsurgical group where patients with higher FTP were kept like Grade 3 (56.6%) and Grade 4 (46.4%). This difference was statistically significant with *P* value of <0.001 as shown in Table 5. Among the surgical group of 22 patients, 77.3% of the patients had only retropalatal obstruction, and in 13.6% of patients retropalatal and retrolingual obstruction was seen; retropalatal, retrolingual, and nasal obstruction was seen in 4.5%; and retropalatal and nasal in 4.5% of patients as shown in Tables 6 and 7. This group of patients was selected for UPPP, and the result of the surgery as defined by 50% reduction in preoperative AHI with postoperative AHI < 20/h was seen to be 95.2%. Significant change in major presenting symptoms was documented six months after surgery as shown in Table 9. Postoperative change in AHI done after 6-month interval was seen to be statistically significant with *P* value < 0.00 as shown in Table 8 and Figure 4. 

## 4. Discussion

Uvulopalatopharyngoplasty is the most common surgical procedure performed for the management of OSAS, but the success rate and the role of UPPP in the management of OSA remain unclear because most studies are limited by small sample size, lack of consensus on a clear definition of surgical success, and an inability to compare UPPP in a blinded manner with CPAP [1, 2, 7]. The main goal of this study was to redefine the ideal clinical parameters to identify those patients with high likelihood of successful UPPP and separate them from those with high likelihood of failure, thus guiding patient selection and improving outcome. Traditionally, a successful outcome of UPPP has been defined as achieving a reduction in AHI of at least 50% and/or a residual AHI of 20 or less. The study format analysing clinical parameters like neck circumference, level/site of obstruction in addition to BMI, tonsil grade, and Friedman palatal position have augmented the guiding criteria for improving the successful result of UPPP in the management of OSAS. Friedman stage I and II were considered for surgery, and stage III was compared as nonsurgical group. Friedman stage was also seen to be significantly correlated with the AHI severity of patient, so the patients in the surgical group were having lesser severity of disease on the basis of AHI as compared to the nonsurgical group. The neck size and BMI of patients in surgical group were seen to be significantly less as compared to nonsurgical group [7, 11, 12]. On the basis of site of obstruction as seen with videoendoscopy with Mueller's manoeuvre, patients with retropalatal and retrolingual were only considered for surgery and all the hypopharyngeal and multilevel obstruction patients were excluded to increase surgical outcome rates. So the videoendoscopy is a complementary diagnostic tool that can be easily performed, especially for surgeons who need to know where and how the obstruction occurs [3, 7, 13]. The successful outcome of the surgery as defined by 50% reduction in preoperative AHI with postoperative AHI < 20/h was seen to be 95.2% as shown in Figure 4. In almost all previous studies done for UPPP, utmost 80% successful treatment outcome was achieved as in all these level/site of obstruction was neglected. As most of the patients have multilevel obstruction with hypopharyngeal as one of the component, UPPP that corrects retropalatal and retrolingual obstruction only is not sufficient treatment. This improved successful treatment goal with UPPP is possible only through proper selection of patients on merits of neck size and site of obstruction in addition to Friedman staging system [7, 8, 11, 13]. In addition, there was no craniofacial abnormality in our selected group, hypopharyngeal obstruction was not considered, and the sample size, once stratified, was relatively small. One of the strengths of the our study is the assessment of pre-UPPP and post-UPPP symptomatology changes in major symptoms as measured by working questionnaires which may strengthen interpretation of the surgical results. Significant changes were noted in major symptoms after surgery. While success rates are slightly higher than those published by Friedman and colleagues, the response seen with this anatomic staging system suggests that this is an effective method for stratifying surgical OSA patients for possible successful UPPP surgery.

## 5. Conclusion

 This study redefines the clinical assessment parameters of OSAS patients for successful outcome of the UPPP. UPPP is a better option for management of obstructive sleep apnoea syndrome in properly selected patients on the basis of Friedman stage and site of obstruction detected by videoendoscopy with muller's maneuver. All cases of obstruction at the palatal level can be addressed by UPPP with satisfactory success rate.

## Figures and Tables

**Table 1:** Age wise distribution of patients (*n* = 50).

Age (year)	*n*	% age
≤30	4	8.0
31 to 40	12	24.0
41 to 50	22	44.0
51 to 60	12	24.0
mean ± SD	44.4 ± 9.3 (18, 60)	

**Table 2:** Site of obstruction seen by videoendoscopy with Muller's maneuver.

Site of obstruction	No. of patients	Percentage
Hypopharyngeal	8	16
Retropalatal	18	36
Hypopharyngeal + retropalatal	6	12
Hypopharyngeal + retrolingual	13	26
Retropalatal + retrolingual	3	6
Retropalatal + nasal	1	2
Retropalatal + retrolingual + nasal	1	2

Total	50	100

**Table 3:** Comparative analysis of surgical and nonsurgical groups on physical parameters.

Physical parameter	Surgical group Mean ± SD	Non surgical group Mean ± SD	*P* value
Neck size cm	32.2 ± 3.2	40.0 ± 1.9	<0.001 (Sig)
BMI (wt/h m^2^)	31.7 ± 2.9	37.0 ± 1.8	<0.000 (Sig)

**Table 4:** Comparison of surgical and nonsurgical group on the basis of tonsil grade.

Tonsil grade	Surgical group (*n* = 22)		Non surgical group (*n* = 28)		*P* value
	*n*	% age	*n*	% age	
0	0	0.0	6	21.4	<0.001 (sig)
1	0	0.0	13	46.4	
2	6	27.3	9	32.1	
3	12	54.5	0	0.0	
4	4	18.2	0	0.0	

*χ* ^2^ test analysis.

**Table 5:** Comparison of surgical and non surgical patient groups on the basis of Friedman tongue position.

FTP	Surgical group (*n* = 22)		Non surgical group (*n* = 28)		*P* value
	*n*	%	*n*	%	
2	12	54.5	0	0.00	<0.001 (sig)
3	10	45.5	15	56.6	
4	0	0.00	13	46.4	

Total	22	100	28	100	

*χ* ^2^ test analysis.

**Table 6:** Distribution of patients in surgical group on site of obstruction (*n* = 22).

Site of obstruction	No. of patients	Percentage
Retropalatal	17	77.3
Retropalatal + retrolingual	3	13.6
Retropalatal + retrolingual	1	4.5
Retropalatal + retrolingual + nasal	1	4.5

Total	22	100

**Table 7:** Distribution of patients on site of obstruction surgical versus nonsurgical groups.

Site of obstruction	Surgical group		Non surgical group		*P* value
	*n*	Percentage	*n*	Percentage	
Single	17	77.3	9	32.1	0.002 (sig)
Multiple	5	22.7	19	67.9	

Total	22	100	28	100	

*χ* ^2^ test analysis.

**Table 8:** Preoperative and postoperative AHI of surgical group.

	Mean	SD	*P* value
Pre-Op PSG AHI score/Hr	43.1	16.4	<0.001 (Sig)
Post-Op PSG AHI score/Hr	13.2	4.1	

**Table 9:** Preoperative and postoperative comparison of symptoms.

Symptoms	Preoperative		Postoperative		*P* value
	*n*	%	*n*	%	
Snoring	18	81.8	3	13.6	<0.001 (Sig)
EDS	20	90.9	2	9.1	<0.001 (sig)
Disturbed Sleep	18	81.8	4	18.2	<0.001 (Sig)
Morning Headache	9	40.9	2	9.1	0.020 (Sig)
Forgetfulness	8	36.4	4	18.2	0.157 (NS)
Dry Mouth	11	50.0	4	18.2	0.008 (Sig)
